# Efficacy of Tai Chi-Style Multi-Component Exercise on Frontal-Related Cognition and Physical Health in Elderly With Amnestic Mild Cognitive Impairment

**DOI:** 10.3389/fragi.2021.636390

**Published:** 2021-04-16

**Authors:** Shao-Yun Yang, Hsuei-Chen Lee, Chih-Mao Huang, Jin-Jong Chen

**Affiliations:** ^1^ Department of Physical Therapy and Assistive Technology, National Yang Ming Chiao Tung University, Taipei, Taiwan; ^2^ Sports and Health Science Research Center, National Yang Ming Chiao Tung University, Taipei, Taiwan; ^3^ Department of Biological Science and Technology, National Yang Ming Chiao Tung University, Hsinchu, Taiwan; ^4^ Center for Intelligent Drug Systems and Smart Bio-devices (IDS^2^B), National Yang Ming Chiao Tung University, Hsinchu, Taiwan; ^5^ Innovation Center of Artificial Intelligence for Precision Exercise and Health Promotion, Yuanpei University of Medical Technology, Hsinchu, Taiwan

**Keywords:** amnestic MCI, Tai-Chi exercise, multi-components exercise, neurocognitive function, functional fitness

## Abstract

Early prevention from accelerated neurocognitive declines with advanced aging and the delay of the onset of dementia have became paramount for the achievement of active aging. The present study examined whether the proposed non-pharmaceutical, multi-component exercise training which combined Tai-Chi exercise, Aerobic fitness, and thera-band therapy protects against age-related neurocognitive and physical deterioration in the older participants with amnestic mild cognitive impairment (aMCI). Participants with aMCI in the quasi-experimental design were assigned to the multi-component exercise group or care control group. Evaluations of neuropsychological function and functional fitness were performed before and after 12-weeks intervention, and after 24-weeks follow-up. Our results showed that the multi-component intervention significantly improved various domains of neurocognitive function, particularly in memory- and frontal-related cognition, and better performance on functional fitness, including muscle strength, cardiopulmonary endurance, and agility. Furthermore, such beneficial effects were preserved after 24 weeks. The findings provide supportive evidence that non-pharmaceutically multi-component intervention with Tai-Chi style practice as a core exercise may protect against age-related neurocognitive and physical deficits and lay the path on developing age-friendly intervention programs to delay, or even reverse, the progression of MCI to dementia.

## Introduction

In the last few decades, the phenomenon of global aging and longevity represents varied aspects of social, economic, and medical challenges. The global population aged 60 years or over numbered 962 million in 2017 and is estimated to double by 2050. The rising cost of mental health and medical care from age-related neurological diseases and neurocognitive declines with population aging, such as dementia, which affects more than 35 million people worldwide, has become a profound burden for the society. Dementia is the most common neurodegenerative disease accompanied by global and specific cognitive impairment including memory, language, and attention, in those over the age of 60. The major neuropsychological conditions in the early stage of dementia usually start from memory, visuo-spatial and language deficits, and continue on to problems of judgment and reasoning (Burns and Iliffe, 2009). Due to the present lack of disease-modifying treatment for dementia, early prevention becomes paramount in order to reduce subsequent disability and achieve better outcomes for aging (Rowe and Kahn, 1997). Mild Cognitive Impairment (MCI) refers to a heterogeneous cognitive impairment with mild deficits in a variety of domains of cognition that has a high conversion rate to dementia. Among MCI patients, amnestic mild cognitive impairment (aMCI) denotes a subtype of MCI associated with memory loss that specifically represents a high risk state for developing dementia. The aMCI-dementia conversion rate is 10–15% per year, compounding to 80% after 6 years (Petersen, 2000; Petersen et al., 2001). Previous behavioral studies had identified that patients with aMCI showed declined neurocognitive function associated with episodic memory, including immediate and delayed recall, and such poor performance in memory significantly correlated with reduced hippocampal volume compared with healthy older adults (Marchiani et al., 2008; Salmon, 2012; Gainotti et al., 2014). The failure of these basic memory functions for older adults lead to the difficulties in maintaining activities of daily living, the subsequent loss of independence, and the reduction of physical as well psychological health. It has been suggested that patients with aMCI are in transitional state between healthy aging and dementia, and are considered to be a critical target for prevention of, and protection from, dementia (Petersen et al., 1999).

Recent cross-sectional and longitudinal studies reported that moderate exercise and physical activity, such as aerobic exercise, can improve age-related cognitive declines, reduce the risk of dementia, and protect against neurocognitive changes related to neurodegenerative diseases such as dementia (Ahlskoget al., 2011; Hoffmann et al., 2016; Öhman et al., 2016). There is also evidence that various types of physical exercise appear to influence different aspects of neurocognitive function. For example, in a systematic review, Sáez de Asteasu et al. (2017) demonstrated that aerobic exercise and resistance training could significantly improved individual’s performance on memory and executive function. Moreover, multi-components exercise showed beneficial effects on memory, executive, verbal skill and general cognition (Sáez de Asteasu, 2017). Some meta-analyses have reported that healthy young and older adults performing aerobic exercise showed improved cognitive function in attention, processing speed, executive function, and memory (Barha et al., 2017; Öhman et al., 2014; Smith et al., 2010). Other meta-analyses, however, failed to demonstrate the positive effects of physical exercise on a variety of measures of neurocognitive performance (Smith et al., 2010). Given the controversial findings that intervention for physical activity alone may not effective to enhance and/or maintain age-related declines in cognitive performance, a multi-component exercise with more structuralized, personalized, moderate-vigorous intensity of long duration or high frequency could be a more effective intervention platform for adults with and without cognitive impairment (Smith et al., 2010; Kirk-Sanchez and McGough, 2013).

Previous studies on exercise-related interventions mainly emphasized one or two exercise components such as strength and aerobic training that may not be the age-friendly intervention programs for the elderly with and without neurocognitive impairment. Multi-component exercise, combining aerobic exercise, strength training, coordination, postural balance training, and a dual task program, may have beneficial effects on maintaining activities of daily living, such as by reducing falls (Petersen et al., 1999; Ahlskog et al., 2011; Hoffmann et al., 2016). Thus far, few studies have been conducted to examine the potential effects of multi-component exercise interventions on neurocognitive function for the elderly with and without cognitive impairment (Smith et al., 2010; Suzuki et al., 2012). In one pioneering study, Suzuki et al. (2012) developed a multicomponent exercise program including aerobic exercises, muscle strength training, and postural balance retraining for the elderly with aMCI. They reported that the elderly with aMCI showed improved neurocognitive function including general cognition, memory of immediate recall, and language ability after training. The findings suggest that the multi-component exercise training could be an effective intervention platform and have potential to preserve memory-related cognition for older individuals with deficits in memory domains (Ahlskog et al., 2011; Sáez de Asteasu et al., 2017).

Tai-Chi exercise is a form of multi-component exercise and an alternative modality of physical activity that may have the potential to improve mental and physical health in older adults (Chang Y. K. et al., 2010; Jahnke et al., 2010). Tai-Chi is a multimodal mind-body exercise regimen that combines mental training and physical exercise and emphasizes motor control and attention during its performance. Previous studies suggested that of the practice of Tai-Chi improves overall mental and physical health in the elderly by reducing pain, stress, and blood pressure, and improving physical fitness, well-being, and quality of life in the elderly (Wang et al., 2010; Holly and Helen, 2012; Hackney and Wolf, 2014). There is also evidence that the exercise of Tai-Chi might be associated with the improvement of several fundamental aspects of cognitive functions, including long-term memory, executive function, attention, and visuospatial function (Miller and Taylor-Piliae, 2014; Wayne et al., 2014). In addition, recent studies suggested that Tai-Chi exercise could slow age-related mental decline, reduce the disease-related risks of neurocognitive impairment, and enhance cognitive performance in elderly individuals with MCI (Man et al., 2010; Lam et al., 2011; Nguyen and Kruse, 2012). Given the Tai-Chi style exercise involve the attentional-control for visuo-spatial coordination, decision-making for planned posture, and executive processing of sequential body movement during practice, we speculated that sustained Tai-Chi exercise may serve as a cognitively stimulating activity to improve frontal-related cognition in the patients with amnestic MCI caused by aging (Kasai et al., 2010; Wei et al., 2013).

The present study aimed to examine t whether the proposed non-pharmaceutical, multi-component exercise training protects against age-related neurocognitive and physical deterioration the older participants with amnestic mild cognitive impairment (aMCI), a transitional state between healthy aging and dementia with specific deficit in memory domain. Specifically, this proposed multicomponent exercise intervention which combined Tai-Chi exercise, Aerobic fitness, and thera-band therapy was designed to evaluate a variety of neurocognitive functions and functional fitness in the older participants with aMCI before and after the 12 weeks intervention, and again at a follow-up 24 weeks after the intervention period. Moreover, to develop a more comprehensive exercise program for the aMCI population which show heterogeneous cognitive impairment in a variety of cognition, the Tai-Chi style multicomponent exercise was designed with 1) aerobic fitness sessions to enhance exercise intensity, 2) with Thera Band session to train upper extremity strength, and 3) with Tai-Chi mind-body exercise to emphasize motor control and attention during its performance.

Given the limited studies developing multicomponent exercise training for the patients with MCI and reported the beneficial effects on memory-related cognition, the proposed non-pharmaceutical intervention additionally developed Tai-Chi style practice as a core exercise and hypothesize that Tai-Chi style multi-component may contribute to the maintenance and/or improvement of frontal-related cognition in the patients with amnestic MCI caused by aging.

## Materials and Methods

### Participants

This study was a quasi-experimental design and used a purposive sampling method to recruit subjects. A total of 202 individuals aged 65 years or older were screened for aMCI in a community senior housing. A total 62 elderly individuals met the criteria of aMCI (32.17%). All participants met Peterson’s criteria of amnestic MCI that includes the following: an objective or subjective memory complaint (determined by CCVLT 10-min recall scores; normal general cognition (MoCA >24 and MMSE ≥24); normal ADL/IADL; and no dementia (Petersen et al., 1999; Tsai et al., 2012; Christa Maree Stephan et al., 2013). Of these, 59 participants with aMCI were recruited to this study and the others refused to participate. Participants were randomly assigned to the multi-component exercise group (MEG; n = 22) or the care control group (CCG; n = 20). All participants provided written informed consent before participation. This study was approved by the Institutional Review Board (IRB) for ethical human subject research, and informed consent was obtained from all participants. Six participants in MEG and one in CCG were excluded after the 12-weeks intervention due to the failure of scheduling for participation. After 24 weeks of follow-up, three participants in MEG and one participant in CCG was dropped. Ultimately, the data of nineteen subjects in MEG (MEG; n = 19; mean age, 83.16 ± 5.7 years; mean length of education, 7.68 ± 4.3 years) and seventeen subjects in CCG (CCG; n = 17; mean age, 82.06 ± 5.5 years; mean length of education, 8.47 ± 5.7 years) were analyzed by SPSS. Exclusion criteria were as follows: failure to meet Petersen’s criteria; neurological or psychiatric disease (GDS >15); and severe musculoskeletal or cardiovascular disease.

### Study Design and Procedures

**FIGURE 1:**
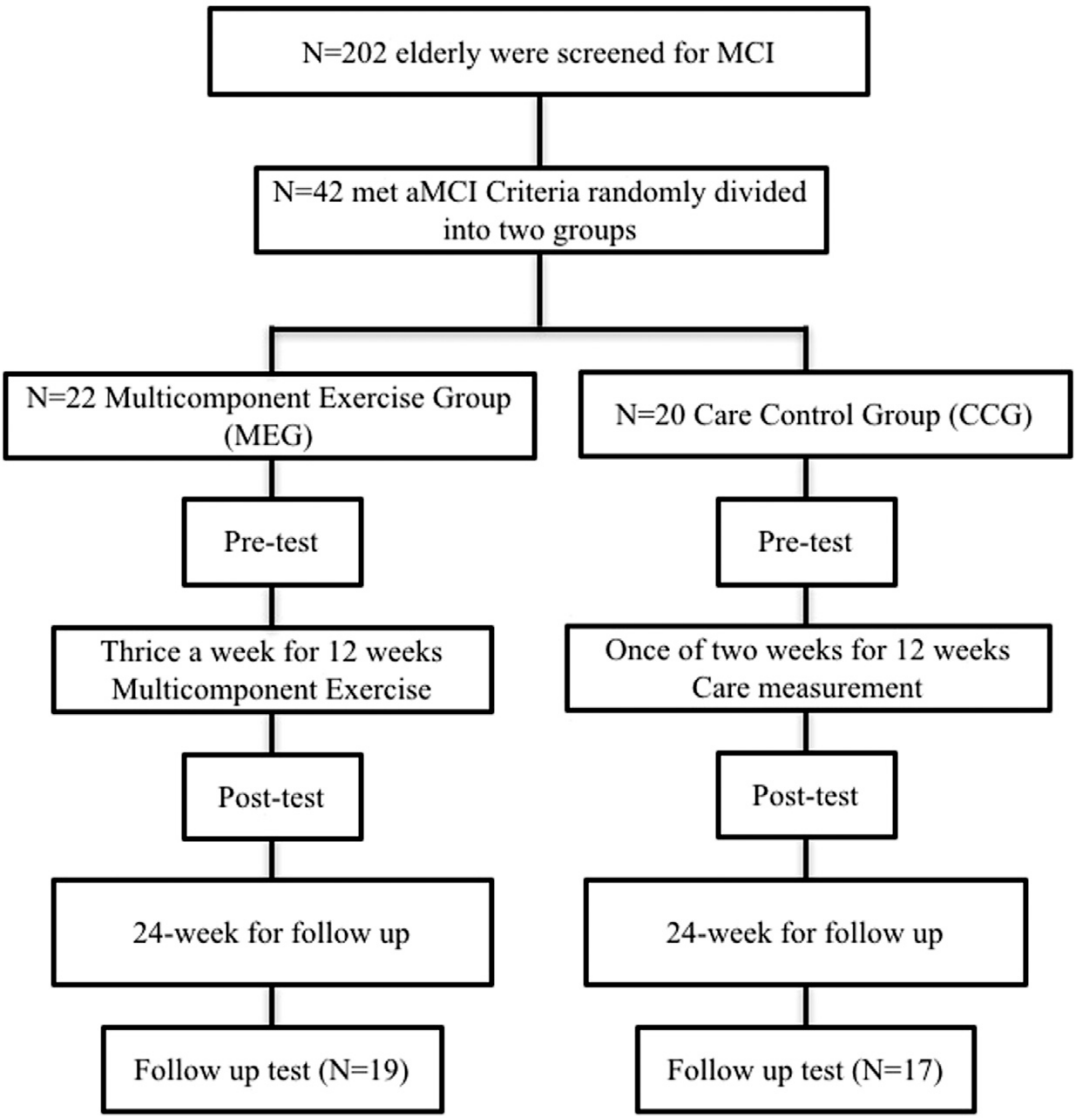
The outline of the participants flow from first contact to study completion.

The behavioral performance on neurocognitive functions and functional fitness for the MEG and CCG were evaluated in three phases: pre-test, 12-weeks post-test and 24 weeks follow-up test. All participants were administered a battery of neuropsychological tests and functional fitness tests before, after the intervention and at the end of the follow-up. Figure 1 shows the study timeline and consort participant flow diagram.

### Intervention

The MEG received exercise intervention for the duration of 60 min, thrice-a-week, over 12 weeks. A more comprehensive Tai-Chi style multi-component exercise program was designed for the elderly with and without aMCI. The exercise program focused on distal joints, such as fingers activated at the proximal joint; with movements emphasizing reciprocal motion and crossing the midline. Aerobic fitness and thera band training were included to increase exercise intensity and muscle strength. Training sessions were comprised of a warm up phase (10 min), aerobic fitness combined with thera band training (15 min), Tai-Chi exercise (25 min), and a cool down phase (10 min). Before and after each exercise session, blood pressure was monitored for all participants to ensure safety and RPE (Ahlskog et al., 2011; Hoffmann et al., 2016; Öhman et al., 2016) was recorded after each exercise intervention. One physiotherapist involved in geriatric rehabilitation and two well-trained instructors conducted each exercise intervention.

The CCG did not receive specific exercise training during the 12-week period. All patients in the CCG received regularly monitoring of blood pressure and body weight, and blood glucose was tested once every 2 weeks during the 12-week period. After the 12-weeks intervention period, the physical activity of both groups were assessed once every 2 weeks during the 24-weeks follow-up period.

### Outcome Measures

The neurocognitive function and functional fitness tests before and after 12-weeks intervention period, and after a 24-weeks follow-up were measured in all older participants with aMCI.

#### Neurocognitive Function Assessment

The Montreal Cognitive Assessment-Taiwan version (MoCA-T) was administrated as a general cognitive function to screen for mild cognitive impairment (Tsai et al., 2012). Seven domains of cognition were included: attention, executive function (abstraction), memory (immediate and delayed recall), verbal ability (fluency and naming), visuo-spatial construction skill, calculation, and orientation. A cutoff point of 24/30 (sensitivity, 92%; specificity, 78%) was posited (Tsai et al., 2012). An additional point was added to the total score in participants with less than 12 years of education. The Chinese California Verbal Learning Test (CCVLT) (Chang C. C. et al., 2010), Mini-Mental State Examination (MMSE) (Folstein et al., 1975), and Geriatric Depression Scale (GDS) (Sheikh and Yesavage, 1986) were used to confirm the type of amnestic MCI. Cutoff points used in this study were six for the CCVLT, 24 for the MMSE, and 15 for the GDS (Chen et al., 2011).

All participants were administrated a battery of neuropsychological tests to assess each individual’s generally cognitive ability. To test memory, participants completed the Logical Memory subtest of Wechsler Memory Scale-Revised. Participants were instructed to remember two short stories (Story A and B) and recall the details of each story immediately and delayed after 25 min. Memory retention and recognition were also assessed (Wechsler, 1987). The Visual Reproduction subtest of WMS-R was used to measure the ability of short-term memory function (Wechsler, 1987). Working memory was assessed via the Corsi Blocks Forward and Backward tasks and Digit-span Forward and Backward tasks in Wechsler Memory Scale, third Edition (WMS-III) to identify different aspects of working memory (Wechsler, 1997). Language ability was assessed using a verbal fluency task in which participants named the categories of colors and animals. Frontal-related executive function was assessed via the Trail Making Test (TMT-A and B) by calculating the time of the task’s completion (Horton and Hartlage, 1994).

#### Functional Fitness Test

The Senior Functional Fitness Test (Rikli and Jones, 1999) was used in this study to assess the levels of functional fitness in several categories: 1) Grip strength (in kilograms), measured by Electronic Handheld Grip Meter (T.K. K5401, Tokyo, Japan). Participants were asked to aqueeze a hand-held dynamometer for 2–3 s. The test was performed twice with the best score recorded for each hand. 2) Walking speed (in meter/second), measured by Usual pace and Fastest pace tests. Participants were asked to walk more than 6 m twice. Usual pace and fastest pace with best score was recorded. 3) Muscle strength, measured by a 30 s stand and sit test as well as a 30 s arm curl test. Participants were asked to perform a test with repeated activities of standing and sitting in a chair for 30 s and to flex their biceps by curling a hand weight for 30 s (5 lb for female, 8 lb for male). 4) Balance, measured by a 30 s open eyes one leg stand test. Participants were asked to stand with their eyes open for 30 s on only their dominant leg with their other leg bent lift and their arms placed on both sides of their body and naturally straight. This test was then repeated using their non-dominant leg. 5) Cardiopulmonary endurance, measured by a 2-min step tests. Participants were asked to perform a 2 min steps test, raising each knee to a point midway between patella and iliac crest. 6) Agility, measured by an 8 ft time up and go test. Participants were asked to quickly stand up from a sitting position and move 8 (2.44 m) twice and their fastest score was recorded. Special annotations were made for participants using a walker or other assistive.

### Statistical Analysis

Statistical analyses were performed using SPSS Statistics 20 (Version 20.0, IBM Corp., Armonk, NY, United States). Descriptive statistics were conducted to present participants’ demographics. The two-sample *t*-test and Chi Square test were conducted to compare basic data, baseline performance of neurocognitive function, and fitness between groups. The intervention effect (main effect and interaction) was assessed using a repeated measures two-way analysis of variance (ANOVA), with Group (MEG, CCG) as a between-subject factor and Time (pre-test, post-test and follow-up test) as a within-subject factor. *p*-values less than 0.05 were considered statistically significant. In addition, *p*-values less than 0.1 were presented to indicate possible trends.

## Results

### Baseline Demographic and Clinical Characteristics

A total of 36 eligible participants were completed all evaluation and assessment of pre-test, post-test, and follow-up-test with 19 in the MEG and 17 in the CCG. Table 1 presents demographic information, for two groups at baseline. No significant differences in gender, age, years of education, or clinical characteristics at baseline were observed between the MEG and CCG.

**TABLE 1:** Participants’ detail demographic characteristics at baseline (Mean ± SD).

Characteristic	Group		*p* value
	MEG	CCG	
N (Male/Female)	19 (3/16)	17 (7/10)	0.139
Age	83.1 ± 5.7	82.0 ± 5.5	0.564
Education	7.6 ± 4.3	8.4 ± 5.7	0.603
Cognitive Function			
MoCA-TS	19.3 ± 2.8	20.5 ± 2.7	0.201
MoCA-MIS	9.0 ± 2.9	9.5 ± 3.4	0.584
MMSE	25.1 ± 1.4	25.4 ± 1.3	0.591
CCVLT	4.7 ± 1.3	4.0 ± 1.6	0.13
GDS	4.1 ± 4.1	5.4 ± 3.8	0.339
Memory-immediately	13.1 ± 6.5	16.9 ± 5.6	0.073
Memory-imm-top	3.1 ± 1.6	4.0 ± 1.1	0.083
Memory-delay	3.5 ± 2.1	5.0 ± 2.4	0.054
Memory-delay-top	2.1 ± 1.4	2.9 ± 1.5	0.116
Memory-retention	52.7 ± 27.0	69.2 ± 24.9	0.066
Memory-recognition	17.7 ± 2.7	19.5 ± 2.7	0.053
Spatial-F	5.3 ± 1.2	5.6 ± 1.6	0.5
Spatial-B	4.4 ± 0.9	5.0 ± 1.3	0.14
Digit-F	10.6 ± 2.0	10.7 ± 1.7	0.907
Digit-B	3.7 ± 1.2	4.1 ± 1.0	0.266
Visual reproduction-TS	39.6 ± 13.8	43.3 ± 17.1	0.483
Verbal-N	10.2 ± 2.4	11.8 ± 3.9	0.146
Verbal-R	1.1 ± 1.3	1.0 ± 1.2	0.725
TMT-A-sec	28.8 ± 18.5	26.5 ± 13.1	0.674
TMT-B-sec	87.7 ± 18.2	73.9 ± 34.5	0.137
Functional Fitness			
Grip-RT	20.1 ± 7.2	22.6 ± 5.4	0.256
Grip-LT	18.8 ± 7.3	20.9 ± 5.0	0.336
6 m-Walk-Usual	0.8 ± 0.2	0.9 ± 0.1	0.336
6 m-Walk-Fastest	1.1 ± 0.2	1.2 ± 0.2	0.336
30 s-stand-sit	12.7 ± 5.7	15.7 ± 4.7	0.108
30 s-arm curl	13.5 ± 4.5	13.8 ± 4.7	0.818
OLS-RT	5.3 ± 3.8	3.6 ± 3.9	0.197
OLS-LT	5.8 ± 4.3	3.9 ± 2.3	0.123
8 ft TUG	9.9 ± 3.3	9.4 ± 3.3	0.629
2 min step	67.0 ± 19.9	63.2 ± 14.8	0.53

MEG, multi-component exercise group; CCG, care control group; MoCA-TS, montreal cognitive assessment- total score; MoCA-MIS, montreal cognitive assessment- memory index score; MMSE, mini-metal state examination; CCVLT, Chinese California verbal learning test; GDS, geriatric depression scale; logical-immediately, logical memory-immediately recall; Logical-imm-top, logical memory-immediately-top score; Logical-delay, logical memory-delay recall; Spatial-F, spatial-forward; Spatial-B, spatial-backward; Digit-F, digit-forward; Digit-B, digit-backward; Visual reproduction-TS, visual reproduction-total score; Verbal-N, verbal-animal-number; Verbal-animal-R, verbal-animal-repeated; OLS-RT and LT, one leg stand- right and left; ft TUG, 8 feet time up and go test.

### Effects of Multi-Component Exercise on Neurocognitive Function

**FIGURE 2:**
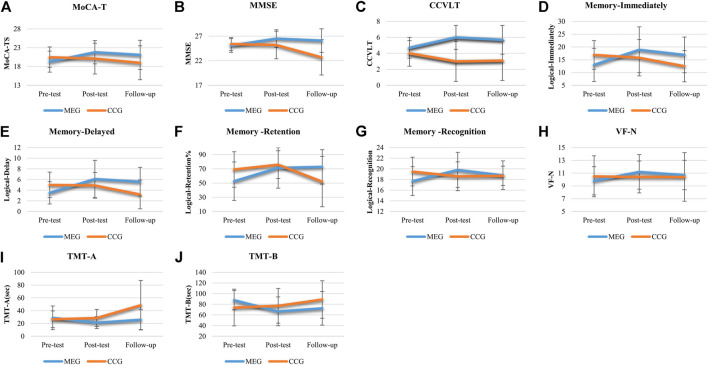
The effects of Tai Chi-style multi-component exercise intervention on cognitive function. The results of cognitive functions revealed significant groups (MEG and CCG) by time (pre-test, post-test and follow up-test) interactions on **(A)** Montreal Cognitive Assessment-Taiwan version (MoCA-T), **(B)** Mini-Mental State Examination (MMSE), **(C)** Chinese California Verbal Learning Test (CCVLT), **(D)** Immediate Recall of Logic Memory (Memory-Immediate), **(E)** Delayed Recall of Logic Memory (Memory-Delayed), **(F)** Retention of Logic Memory (Memory-Retention), **(G)** Recognition of Logic Memory (Memory-Recognition), **(H)** Verbal Fluency-number of naming items (VF-N), **(I)** Trail Making Test-A (TMT-A), and **(J)** Trail Making Test (TMT-B).

The results of the effects of Tai-Chi style multi-component exercise on neurocognitive function are presented in Figure 2; Table 2. The effects of intervention on cognitive outcome measures revealed significant groups (MEG and CCG) by time (pre-test, post-test and follow-up test) interactions (*p* < 0.05) for MoCA-T [*F*_(2,68)_ = 4.535, *p* = 0.014, η^2^ = 0.118], MMSE [*F*_(2,68)_ = 9.755, *p* < 0.001, η^2^ = 0.223], CCVLT [*F*_(2,68)_ = 5.664, *p* = 0.005, η^2^ = 0.143], GDS [*F*_(2,68)_ = 4.042, *p* = 0.022, η^2^ = 0.106], Immediate Recall of Logic Memory (Memory-immediately) [*F*_(2,68)_ = 11.701, *p* < 0.001, η^2^ = 0.256], Immediate Recall of Logic Memory with top score (Memory -imm-top) [*F*_(2,68)_ = 13.448, *p* < 0.001, η^2^ = 0.283], Delay Recall of Logic Memory (Memory - delay) [*F*_(2,68)_ = 15.095, *p* < 0.001, η^2^ = 307], Delay Recall of Logic Memory with top score (Memory -delay-top) [*F*_(2,68)_ = 10.310, *p* < 0.001, η^2^ = 0.233], Retention of Logic Memory (Memory -retention) [*F*_(2,68)_ = 9.133, *p* < 0.001, η^2^ = 0.212], Recognition of Logic Memory (Memory -recognition) [*F*_(2,68)_ = 6.067, *p* = 0.004, η^2^ = 0.151], Verbal Fluency-number of naming items (VF-N) [*F*_(2,68)_ = 3.363, *p* = 0.04, η^2^ = 0.090], Verbal Fluency-number of repeated items (VF-R) [*F*_(2,68)_ = 5.589, *p* = 0.006, η^2^ = 0.141], Trail Making Test-A (TMT-A) [*F*_(2,68)_ = 8.271, *p* = 0.003, η^2^ = 0.196] and Trail Making Test-B (TMT-B) [*F*_(2,68)_ = 8.861, *p* = 0.001, η^2^ = 0.207] (Figure 2; Table 2).

**Table 2:** Intervention effects on cognitive function (Mean ± SD).

Cognitive Function	Pre-tests		Post-tests		Follow up-test		Group by Time interactions
	MEG	CCG	MEG	CCG	MEG	CCG	*p* value
MoCA-TS	19.3 ± 2.8a**	20.5 ± 2.7	21.8 ± 3.1	20.1 ± 4.1	21.1 ± 3.9b*	19.0 ± 4.5	0.014*
MoCA-MIS	9.0 ± 2.9	9.5 ± 3.4	9.7 ± 2.7	10.0 ± 3.1	9.7 ± 2.8	7.9 ± 3.6	0.088
MMSE	25.1 ± 1.4a**	25.4 ± 1.3	26.5 ± 1.8	25.2 ± 2.8c**	26.1 ± 2.4	22.7 ± 3.6b**	<0.001***
CCVLT	4.7 ± 1.3a**	4.0 ± 1.6	6.0 ± 1.5	3.0 ± 2.5	5.7 ± 1.8b*	3.1 ± 2.5	0.005**
GDS	4.1 ± 4.1a*	5.4 ± 3.8	1.7 ± 4.88	4.3 ± 5.5	3.0 ± 3.2	2.8 ± 2.8b**	022*
Memory-immediately	13.1 ± 6.5a***	16.9 ± 5.6	18.9 ± 9.0	15.8 ± 7.1c*	16.9 ± 7.0b*	12.5 ± 6.1b***	<0.001***
Memory-imm-top	3.1 ± 1.6a***	4.0 ± 1.1	4.6 ± 1.4c*	3.8 ± 1.4c*	4.1 ± 1.5b**	3.1 ± 1.4b*	<0.001***
Memory-delay	3.5 ± 2.1a***	5.0 ± 2.4	6.1 ± 3.5	4.9 ± 2.4c**	5.6 ± 2.7b***	3.2 ± 2.7b**	<0.001***
Memory-delay-top	2.1 ± 1.4a***	2.9 ± 1.5	3.7 ± 1.9	3.4 ± 1.5c*	3.7 ± 1.9b***	2.3 ± 2.0	<0.001***
Memory-retention	52.7 ± 27.0a**	69.2 ± 24.9	71.4 ± 28.5	75.9 ± 19.5c**	72.8 ± 24.2b**	52.1 ± 35.5b*	<0.001***
Memory-recognition	17.7 ± 2.7a**	19.5 ± 2.7	19.8 ± 3.3	18.6 ± 2.7c**	18.8 ± 2.7b*	18.7 ± 1.8b*	0.004**
Spatial-F	5.3 ± 1.2	5.6 ± 1.6	6.0 ± 1.8	5.2 ± 1.2	5.4 ± 1.3	5.5 ± 1.2	0.139
Spatial-B	4.4 ± 0.9	5.0 ± 1.3	4.7 ± 1.3	4.9 ± 1.6	4.1 ± 1.2	4.7 ± 1.2	0.516
Digit-F	10.6 ± 2.0	10.7 ± 1.7	11.2 ± 2.7	10.7 ± 2.4	11.6 ± 2.4b**	10.4 ± 2.5	0.414
Digit-B	3.7 ± 1.2a**	4.1 ± 1.0	4.5 ± 1.5	4.1 ± 1.3	4.2 ± 1.5	4.0 ± 1.3	0.104
Visual reproduction-TS	39.6 ± 13.8a**	43.3 ± 17.1	47.8 ± 17.4	45.0 ± 19.5	45.8 ± 14.8	51.0 ± 26.6	0.288
Verbal-N	9.8 ± 2.2a**	10.5 ± 3.2	11.2 ± 2.7	10.4 ± 2.4	10.7 ± 2.3b**	10.4 ± 3.8	0.04*
Verbal-R	1.5 ± 1.4a*	1.1 ± 1.3	0.8 ± 0.8	1.5 ± 1.6	1.2 ± 1.2	1.0 ± 3.8	0.006**
TMT-A-sec	28.8 ± 18.5a*	26.5 ± 13.1	21.2 ± 9.4	28.6 ± 13.4c**	25.9 ± 15.6	48.6 ± 38.7b*	0.003**
TMT-B-sec	87.7 ± 18.2a**	73.9 ± 34.5	66.6 ± 27.0	77.2 ± 32.6c*	72.4 ± 31.5b*	89.0 ± 35.1b*	0.001**

*p* < 0.5a*, *p* < 0.01a**, *p* < 0.001a*** compared between pre-test and post-test within group.

*p* < 0.5b*, *p* < 0.01b**, *p* < 0.001b*** compared between pre-test and follow up-test within group.

*p* < 0.5c*, *p* < 0.01c**, *p* < 0.001c*** compared between post-test and follow up-test within group.

After the 12-weeks intervention, significant improvements in a variety of neurocognitive functions were observed for the MEG, including MoCA-TS [*p* = 0.003], MMSE [*p* = 0.003], CCVLT [*p* = 0.004], GDS [*p* = 0.010], Memory - immediately [*p* < 0.001], Memory -imm-top [*p* < 0.001], Memory - delay [*p* = 0.001], Memory -delay-top [*p* < 0.001], Memory -retention [*p* = 0.005], Memory -recognition [*p* = 0.003], Digit span-Backward [*p* = 0.009], VR-TS [*p* = 0.001], VF-N [*p* = 0.002], VF-R [*p* = 0.01], and TMT-A and TMT-B [*p* = 0.014; *p* = 0.002]. The neurocognitive function of Digit span-Forward, Spatial-Forward and Backward were maintained after 12 weeks (Figure 2; Table 2).

After the 24-weeks follow-up period, significant improvements in neurocognitive function were compared with pre-test for the MEG were observed, including MoCA-T [*p* = 0.021], CCVLT [*p* = 0.045], Memory - immediately [*p* = 0.015], Memory -imm-top [*p* = 0.002], Memory - delay [*p* < 0.001], Memory -delay-top [*p* < 0.001], Memory -retention [*p* = 0.001], Memory -recognition [*p* = 0.047], Digit span-Forward [*p* = 0.006], and TMT-B [*p* = 0.034].

For CCG, none of the neurocognitive function tests showed significant changes after the 12-weeks intervention period. After the 24-weeks follow up period, however, a decline of neurocognitive function was observed compared with post-tests, including MoCA-MIS [*p* = 0.019], MMSE [*p* = 0.001], Memory - immediately [*p* = 0.028], Memory -imm-top [*p* = 0.045], Memory - delay [*p* = 0.007], Memory -delay-top [*p* = 0.018], Memory -retention [*p* = 0.009], Memory -recognition [*p* = 0.009] and TMT-A and TMT-B [*p* = 0.009; *p* = 0.037]. Moreover, the significant declines of neurocognitive function was also observed compared with pre-tests including MMSE [*p* = 0.004], Memory - immediately [*p* = < 0.001], Memory -imm-top [*p* = 0.016], Memory - delay [*p* = 0.002], Memory -retention [*p* = 0.014], Memory -recognition [*p* = 0.014], TMT-A and TMT-B [*p* = 0.010; *p* = 0.020] (Figure 2; Table 2).

### Effects of Multi-Component Exercise on Functional Fitness

**FIGURE 3:**
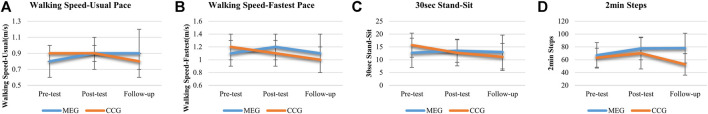
The effects of Tai Chi-style multi-component exercise intervention on functional fitness. The results of physical functional fitness revealed significant groups (MEG and CCG) by time (pre-test, post-test and follow up-test) interactions on **(A)** Walking Speed-Usual Pace test (m/s), **(B)** Walking Speed-Fastest Pace test (m/s), **(C)** 30 s stand and Sit test (30 s Stand-Sit), and **(D)** 2 min Steps test (2 min Steps).

The results of the effects of Tai-Chi style multi-component exercise on functional fitness are presented in Figure 3; Table 3. The effects of multi-component exercise intervention on functional fitness revealed significant group (MEG and CCG) by time (pre-test, post-test and follow-up test) interactions (*p* < 0.05) for walking speed measured by usual pace (m/s) [*F*_(2,68)_ = 3.583, *p* = 0.033, η^2^ = 0.095] and by fastest pace (m/s) [*F*_(2,68)_ = 2.097, *p* = 006, η^2^ = 0.152], 30 s stand and sit test [*F*_(2,68)_ = 4.543, *p* = 0.019, η^2^ = 0.118] and 2 min steps test [*F*_(2,68)_ = 7.004, *p* = 0.002, η^2^ = 0.171] (Figure 3; Table 3).

**Table 3:** Intervention effects on Functional Fitness (Mean±SD)

Functional Fitness	Pre-tests		Post-tests		Follow up-test		Group by Time interactions
	MEG	CCG	MEG	CCG	MEG	CCG	*p* value
Gript-RT	20.1 ± 7.2	22.6 ± 5.4	19.6 ± 6.5	22.1 ± 6.7	19.6 ± 5.5	20.2 ± 4.9b**	0.106
Grip-LT	18.8 ± 7.3	20.9 ± 5.0	18.1 ± 5.8	20.6 ± 5.3c*	18.2 ± 5.8	18.9 ± 4.2b**	0.059
Walk Speed-usual pace (m/s)	0.8 ± 0.2a*	0.9 ± 0.1	0.9 ± 0.2	0.9 ± 0.1c***	0.9 ± 0.3	0.8 ± 0.1	0.033*
Walk Speed-fastest pace (m/s)	1.1 ± 0.2a**	1.2 ± 0.2	1.2 ± 0.2	1.1 ± 0.2c**	1.1 ± 0.3	1.0 ± 1.2b*	0.006**
30 s-stand-sit	12.7 ± 5.7	15.7 ± 4.7a*	13.5 ± 4.5	12.7 ± 5.1	13.0 ± 6.6	11.1 ± 5.3b**	0.019
30 s-arm curl	13.5 ± 4.5a**	13.8 ± 4.7	15.5 ± 3.3	15.9 ± 3.0	15.7 ± 4.8b**	14.3 ± 4.8	0.352
One leg stand- RT	5.3 ± 3.8	3.6 ± 3.9	8.9 ± 8.2c**	4.1 ± 3.7	5.5 ± 7.0	3.2 ± 4.1	0.167
One leg stand- LT	5.8 ± 4.3	3.9 ± 2.3a*	7.5 ± 5.2	2.8 ± 2.8	5.9 ± 6.5	3.2 ± 4.7	0.111
8 ft TUG	9.9 ± 3.3a***	9.4 ± 3.3	8.4 ± 2.4	9.4 ± 2.6c**	9.6 ± 5.1	11.2 ± 3.7b*	0.162
2 min steps	67.0 ± 19.9a*	63.2 ± 14.8	77.6 ± 17.7	70.1 ± 24.4c**	78.1 ± 23.2b*	52.8 ± 16.8b*	0.002**

*p* < 0.5a*, *p* < 0.01a**, *p* < 0.001a*** compared between pre-test and post-test within group.

*p* < 0.5b*, *p* < 0.01b**, *p* < 0.001b*** compared between pre-test and follow up-test within group.

*p* < 0.5c*, *p* < 0.01c**, *p* < 0.001c*** compared between post-test and follow up-test within group.

After 12 weeks intervention period, significant improvements were observed in the MEG for walking speed measured by usual and fastest pace (m/s) [*p* = 0.021; *p* = 001], 30 s arm curl test [*p* = 0.007], 8 ft up and go test (TUG) [*p* < 0.001] and 2 min steps test [*p* = 0.026]. Other measures of functional fitness were maintained at the original level after 12 weeks training sessions. After 24 weeks follow-up period, significant improvements were observed in MEG for 30 s arm curl test [*p* = 0.009] and 2 min steps test [*p* = 0.013] compared with pre-test phase.

For CCG, none of the functional fitness tests showed changes after the 12-weeks intervention period excepted the 30 s stand and sit test [*p* = 0.049] and OLS-L [*p* = 0.012]. However, after the 24-weeks follow-up period, a decline of functional fitness was observed compared with post-tests, including grip strength of left hand (grip-L) [*p* = 0.023], usual and fastest pace of walking speed (m/s) [*p* < 0.001; *p* = 001], 8 ft TUG [*p* = 0.002] and 2 min steps test [*p* = 0.002]. Moreover, the decline of functional fitness was also observed compared with pre-tests including grip-L [*p* = 0.002], fastest pace of walking speed (m/s) [*p* = 029], 30 s arm curl test [*p* = 0.009], 30 s stand and sit test [*p* = 0.009], 8 ft TUG [*p* = 0.036], and 2 min steps test [*p* = 0.040] (Figure 3; Table 3).

## Discussion

Our findings showed the benefits of a Tai-Chi style multi-component exercise intervention for neurocognitive function (particularly in frontal- and memory-related cognition) and functional fitness in older individuals with aMCI. Specifically, the 12-weeks Tai-Chi style multi-component exercise intervention significantly improved several domains of neurocognitive function including general cognition, immediate and delayed recall of memory function, recognition of memory, language production, and frontal-related executive function. Moreover, we found enhanced levels of functional fitness in various aspects, including walking speed, muscle strength, cardiopulmonary endurance, and agility. Finally, significant group indicate a prolonged effect of Tai-Chi style multi-component exercise during the 24-weeks follow-up period by time interactions in neurocognitive function and functional fitness. Such improvement in both neurocognitive and physical function were found in memory recall, retention, and recognition, frontal-related executive function, walking speed, muscle strength, and cardiopulmonary function. Given currently no therapies are available for prevention or treatment of dementia despite the great efforts, the proposed non-pharmaceutical multi-component exercise training with Tai-Chi style practice as a core exercise may provide an effective platform for developing protocol for dementia prevention and therapy.

These findings corroborate previous findings that multi-component exercise programs provide an effective platform and interface to enhance the performance of memory-related cognition and reduce the risk of dementia in aMCI population (Snowden et al., 2011; Suzuki et al., 2012; Zhang et al., 2016). Moreover, given the Tai-Chi style exercise involves the combination of physical and mental stimulating activities during its performance (Nithianantharajah and Hannan, 2006; Mora, 2013), the proposed multicomponent exercise showing beneficial effects on both frontal-related and memory-associated cognition is congruent with the Cognitive Reserve Hypothesis. The Cognitive Reserve Hypothesis has been proposed to suggest that stimulating activities in daily life may help to create a cognitive reserve, a protective mechanism that increases the cognitive and brain capacity to cope with pathologies, such as mild cognitive impairment (MCI) and dementia in the elderly (Stern, 2009). Our results are consistent with this Cognitive Reserve Hypothesis and further suggest that sustained practice in Tai-Chi style multi-component exercise could protect against age-related neurocognitive declines, attenuate neural dysfunction, and postpone the onset of dementia (Stern, 2009, 2012; Huang and Huang, 2019).

Previous studies indicate that age-related declines in episodic memory are the earliest manifestation of, and could be the clinical evaluation of prediction for, progress toward dementia in the aMCI population (Salmon, 2012; Gainotti et al., 2014). We found that the intervention group improved in a variety of aspects of episodic memory that are compromised in aMCI, including immediate recall, delayed recall, retention, and recognition after 12 weeks of Tai-Chi style multi-component exercise and that these beneficial effects lasted for at least 24 weeks. In contrast, the control group, which did not undergo exercise intervention, showed gradually declined cognition in episodic memory. The Tai-Chi style exercise emphasizes the integration among body movement, including limbs switching and motor planning. More importantly, this mind-body exercise also involve the visuo-spatial processing and the execution of sequential information for body coordination during practice. It is quite reasonable to posit that sustained Tai-Chi exercise may serve as a cognitively stimulating activity to protect against age-related neurocognitive deterioration. Our findings are consistent with the previous finding showing a preserved memory function in the female patients with MCI (Kasai et al., 2010). Finally, we further provide supportive evidence that the characteristics of Tai-Chi exercise, including concentration, and memorization of sequences of movements, maintaining the focus on body consciousness and movement planning may contribute to improve and scaffold memory function in elderly with amnestic MCI caused by aging (Kasai et al., 2010; Wei et al., 2013).

Our results showed that Tai-Chi style multi-component exercise significantly improves the efficiency of executive function compared with the control group. Similarly, previous study reported that Tai-Chi training for 15 weeks is associated with a significant improvement in executive function measured by TMT in the elderly with aMCI (Sungkarat et al., 2017). Moreover, Wei et al. (2013) found that, during practice of Tai-Chi, when middle-aged adults were instructed to control breath, focus on concentration, perform mindfulness, and inhibit distractions from surroundings, participants showed better performance on executive control. They concluded that the beneficial effects could stem from the features of Tai-Chi that requires gross motor skills, such as moving the trunk and limbs in 3-dimensional space, resulting in the simultaneously integration of multiple sources of spatial information from proprioception (Wei et al., 2013). Chang Y. K. et al. (2010) posited that several potential mediators between exercise and cognition, including physical resources, disease states, and mental resources, play indirect or direct roles between physical activity and cognition. The beneficial effects of executive functions observed on the patients with aMCI in this study may be attributable to the direct brain-behavioral mediators and indirect disease-states mediator of the association between exercise and cognition. These converging findings suggest that Tai-Chi, as a mind-body exercise, involves the learning and recall of movement patterns, is associated with sustained attentional focus, and engages working memory, task-switching, cognitive flexibility, and executive functions (Zheng, et al., 2015b). In keeping with the above research, our results provide supportive evidence that Tai-Chi style multi-component exercise could be an effective and comprehensive exercise program for the aMCI population as it leads to amelioration of impairment in a variety of cognitive domains.

We reported that a 12 weeks Tai-Chi style multi-component exercise program also showed improved levels of functional fitness, including walking speed, muscle endurance of upper limbs, integrity skill, and cardiopulmonary function, and that such benefits were maintained after intervention in patients with aMCI. The results were consistent with previous literature showing that Tai-Chi could improve walking speed, integraity skill (TUG), and cardiopulmonary function in the healthy elderly (Sun et al., 2015; Zheng et al., 2015a). For example, Lin et al. (2015) demonstrated that 16 weeks of Tai-Chi in conjunction with thera-band resistance exercise displayed a significant increase in muscle strength of upper and lower extremities, integrity skill (TUG) and cardiopulmonary function in community-dwelling older participants. Moreover, they speculate that the effects of exercise on lower extremities, integrity skill, and cardiopulmonary function could stem from Tai-Chi exercise, and the effects of exercise on upper extremities could stem from thera-band resistance exercise (Lin et al., 2015).

There is converging evidence showing that the ability to employ lower extremities (e.g., walking speed and TUG) appears to be associated with the performance of cognitive function and that walking speed can be a predictor of the risk of age-related neurocognitive decline. For instance, Kikkert et al. (2016) demonstrated that the slowing of walking speed (0.68–1.1 m/s) preceded cognitive decline and can serve as an effective markers to predict the rates of cognitive decline (Eggermont et al., 2010; Kikkert et al., 2016). Our results show that a 12 weeks Tai-Chi style multi-component exercise led to a significant improvement in the fitness level of walking speed, suggesting a benefit of reduced risk of developing dementia for the aMCI population. In addition, it has been suggested that better cardiopulmonary function relates to better cognitive function; higher V̇O_2_ max is associated with better general cognition, memory function, executive function, and motor skill. Our results are consistent with the previous findings and further suggest that multi-component exercise that includes Tai-Chi exercise, thera band training, and aerobic fitness could provide an effective and comprehensive intervention program to enhance the physical activity and functional fitness of the aMCI population. Further studies are encouraged to explore whether the beneficial effects of Tai-Chi style multi-component exercise can be observed in patients with age-related neurodegenerative diseases, such as dementia, and in patients suffering from physical frailty (Chen et al., 2015).

Despite the results demonstrating improved neurocognition and fitness following 12 weeks of a Tai-Chi style multi-component exercise, the present study had some limitations. First, the sample size was relatively small. Second, the gender ratio of participants is predominantly female, which should be balance to represent the general pattern of the aMCI population. Third, the age range of participants is relatively old in this study. Particularly challenging is the length of the follow-up period. Previous studies have indicated that long follow-up periods may correct for the fluctuation stage of aMCI.

## Conclusion

In conclusion, this study provides supportive evidence for the beneficial effects of a 12-weeks Tai-Chi style multi-component exercise intervention on frontal- and memory-related neurocognitive function and functional fitness in the elderly with aMCI, a transitional state between healthy aging and dementia. Moreover, prolonged effects of Tai-Chi style multi-component exercise after a 24-weeks intervention on recall and recognition of memory function, frontal-related executive function, and walking speed, muscle strength, as well as cardiopulmonary function were reported. We concluded that older adults with neurocognitive impairment could be benefit from the age-friendly Tai-Chi style multi-component exercise training on mental, cognitive, and physical health to improve their neurocognitive as well physical ability to protect against deficits related to neurodegenerative diseases and to scaffold effective function while maintaining high levels of engagement in daily life. Given currently no therapies are available for prevention or treatment of dementia despite the great efforts, the proposed non-pharmaceutical multi-component exercise training with Tai-Chi style practice as a core exercise provide an effective platform for developing protocol for dementia prevention and therapy.

## Data Availability

The original contributions presented in the study are included in the article/Supplementary Material, further inquiries can be directed to the corresponding authors.
